# Effects of the *DRD4* −521 C/T SNP on Local Neural Activity and Functional Connectivity in Children With ADHD

**DOI:** 10.3389/fpsyt.2021.785464

**Published:** 2022-01-06

**Authors:** Huan Zhang, Binrang Yang, Gang Peng, Linlin Zhang, Diangang Fang

**Affiliations:** ^1^Department of Zunyi Medical University Zhuhai, Zhuhai, China; ^2^Centre for Child Care and Mental Health, Shenzhen Children's Hospital, Shenzhen, China; ^3^Department of Adolescent Gynecology, Shenzhen Children's Hospital, Shenzhen, China; ^4^Department of Radiology, Shenzhen Children's Hospital, Shenzhen, China

**Keywords:** ADHD, *DRD4* −521 C/T SNP, regional homogeneity, fractional amplitude low-frequency fluctuation, functional connectivity

## Abstract

**Objective:** The present study aimed to investigate the effects of the dopamine receptor D4 (*DRD4*) −521 C/T single-nucleotide polymorphism on brain function among children with attention deficit hyperactivity disorder (ADHD) and to evaluate whether brain function is associated with behavioral performance among this demographic.

**Methods:** Using regional homogeneity, fractional amplitude low-frequency fluctuation, and functional connectivity as measurement indices, we compared differences in resting-state brain function between 34 boys with ADHD in the TT homozygous group and 37 boys with ADHD in the C-allele carrier group. The Conners' Parent Rating Scale, the SNAP-IV Rating Scale, the Stroop Color Word Test, the go/no-go task, the n-back task, and the working memory index within the Wechsler Intelligence Scale for Children-Fourth Edition were selected as comparative indicators in order to test effects on behavioral performance.

**Results:** We found that TT homozygotes had low behavioral performance as compared with C-allele carriers. The regional homogeneity for TT homozygotes decreased in the right middle occipital gyrus and increased in the right superior frontal gyrus as compared with C-allele carriers. In addition, the right middle occipital gyrus and the right superior frontal gyrus were used as the seeds of functional connectivity, and we found that the functional connectivity between the right middle occipital gyrus and the right cerebellum decreased, as did the functional connectivity between the right superior frontal gyrus and the angular gyrus. No statistically significant differences were observed in the respective brain regions when comparing the fractional amplitudes for low-frequency fluctuation between the two groups. Correlation analyses demonstrated that the fractional amplitude low-frequency fluctuation in the precentral gyrus for TT homozygotes were statistically significantly correlated with working memory.

**Conclusions:** We found differing effects of *DRD4* −521 C/T polymorphisms on brain function among boys with ADHD. These findings promote our understanding of the genetic basis for neurobiological differences observed among children with ADHD, but they must be confirmed in larger samples.

## Introduction

Attention deficit hyperactivity disorder (ADHD) is a common neurodevelopmental disorder characterized by age-inappropriate inattention, hyperactivity, and impulsivity (1). ADHD has a worldwide prevalence rate of ~7.2% (2), with a corresponding prevalence rate of 5.6% in China (3). Male-to-female sex ratios are reported in the range of 2:1 to 4:1 (4). Symptoms persist into adulthood in ~60% of children with ADHD (5). ADHD is usually associated with a variety of negative outcomes, including high dropout rates, social barriers, criminal behaviors, and professional failures, which may have serious impacts on individuals, families, and society (6).

Previous studies have shown that ADHD has high heritability (7). A promising candidate gene for ADHD is the gene encoding dopamine receptor D4 (*DRD4*), which is mapped to the short arm of chromosome 11 located at 11p15.5 (8). *DRD4* mediates the post-synaptic activity of dopamine and participates in cognitive functions and emotional responses, including attention, perception, planning, language, and memory (9–11). The −521 C/T single-nucleotide polymorphism (SNP), located 521 bp upstream of the transcription start site for *DRD4*, is responsible for the regulation of the transcription rate for this gene. Studies have shown that the *DRD4* −521 C/T SNP is associated with specific personality traits (12), novelty seeking, schizophrenia risk (13), cognitive impairment (i.e., speech fluency and working memory) (14), and executive dysfunction (15). *DRD4* −521 C/T polymorphisms can adjust transcription initiation frequency by changing the affinity of the *DRD4* mRNA polymerase and the respective promoter in order to increase or decrease DRD4 expression levels. A previous study reported that the transcriptional activity for the T allele in the *DRD4* −521 C/T SNP was 40% lower than that of the C allele (16). Additionally, a case-control study found that the frequency of the T allele in children with ADHD was statistically significantly higher than that of the C allele, while the frequencies of the C and T alleles in the children's neurotypical counterparts were similar (17). Therefore, based on findings within the literature to date, the T allele is considered a risk gene for ADHD. Drug therapy has proven that reductions in the dopamine neurotransmitter contribute to the etiology of ADHD. Methylphenidate acts to improve the symptoms of inattention and hyperactivity *via* the pharmacological mechanism of increasing dopamine levels from the synaptic cleft by reducing dopamine reuptake and prolonging its binding time to receptors (18–20). Moreover, the *DRD4* −521 C/T SNP has been confirmed to be a critical factor in the pathogenesis of ADHD (17, 21, 22).

Functional magnetic resonance imaging (fMRI) is becoming an increasingly common approach for understanding the pathological mechanisms mediating ADHD risk (23). Resting-state functional magnetic resonance imaging (rs-fMRI) is widely used in neuropsychological research because of its high resolution and lack of radiation; this imaging modality can more sensitively reflect differences in brain function as compared with neuropsychological tests (24). To date, there has only been one imaging study regarding the *DRD4* −521 C/T SNP. That study found that C-allele carriers and those with CC homozygous genotypes had enhanced memory functionality with respect to novel perception and salient stimulation as compared to participants with TT genotypes, which may be mediated *via* activation of the ventral striatum and hippocampus through variations in this genotype (25). At present, most research has focused on the impact of polymorphisms in the 48-bp (base pair) variable-number tandem repeat (VNTR) region in exon 3 of the *DRD4* on brain functions in children with ADHD (26–28). There have been relatively few studies on the association between the *DRD4* −521 C/T SNP in the promoter of the non-coding region for the *DRD4* and ADHD risk and outcomes. The potential effects of this polymorphism on ADHD brain function are currently unclear.

In the current study, we investigated the effects of the *DRD4* −521 C/T SNP on brain function in boys with ADHD. The relationship between brain function and behavioral performance was also explored. Brain imaging data from 71 children with ADHD were acquired using magnetic resonance scanning. The participants were divided into TT homozygous and C-allele carrier groups according to genotype. Brain indicators, such as regional homogeneity (ReHo), fractional amplitude low-frequency fluctuation (fALFF), and functional connectivity (FC), were calculated in order to detect potential differences between the two groups. Behavioral performance was assessed using the Conners' Parent Rating Scale (CPRS), the SNAP-IV Rating Scale, the Stroop Color Word Test (SCWT), the go/no-go task, the n-back task, and the working memory index (WMI) in the Wechsler Intelligence Scale for Children-Fourth Edition (WISC-IV) in order to test the multidimensional abilities of children with ADHD. Based on previous findings, we hypothesized that TT homozygotes would have lower levels of spontaneous neuronal activity and FC as compared with C-allele carriers. The abilities of children with the TT homozygous genotype with respect to behavioral performance were worse as compared with C-allele carriers in prior research, and there is an established correlation between behavioral performance and brain function in general.

## Materials and Methods

### Participants

Seventy-one participants were recruited from the Children's Care and Mental Health Center at Shenzhen Children's Hospital. Eligible participants were diagnosed by experienced pediatricians using the Diagnostic and Statistical Manual of Mental Disorders, 4th Revision (DSM-IV). The children and their parents were interviewed *via* the Kiddie-Schedule for Affective Disorders and Schizophrenia–Present and Lifetime Version (K-SADS-PL). The inclusion criteria were as follows: (1) aged between 8 and 9 years; (2) a full-scale IQ (FSIQ) above 70 as assessed by the WISC-IV; (3) normal vision and hearing; and (4) right-handedness. The exclusion criteria were as follows: (1) such as learning disabilities, tic disorders, conduct disorders, anxiety, depression, and other mental disorders; (2) ADHD medication, behavioral training, psychotherapy, and other treatments; and (3) metal objects that are difficult to remove (i.e., tooth implants). Ethics approval was obtained from the Medical Research Ethics Committee at Shenzhen Children's Hospital. Written informed consent was obtained from all the participants and their parents.

### Genotyping

Peripheral venous blood was collected from the participants. Genomic DNA was extracted from whole blood using a Flexi Gene DNA kit (QIAGEN, Hilden, Germany). PCR amplification was performed following DNA extraction. Participants were divided into TT homozygous genotypes (TT homozygous group, *n* = 34) and C-allele carriers (C-allele carrier group, *n* = 37; TC genotype = 29, CC genotype = 8) based on genotypes detected *via* agarose gel electrophoresis.

### Measurements

#### ADHD Symptoms

The SNAP-IV Rating Scale is mainly used for ADHD screening, auxiliary diagnosis, evaluating intelligence efficacy, and evaluating symptom improvement in children and adolescents aged 6–18 years. This scale contains 26 items, which are divided into three subscales: inattention (IA), hyperactivity/impulsivity (HI), and oppositional defiant disorder (ODD). Each item uses a 4-point Likert scale: 0 for “not at all,” 1 for “a little bit,” 2 for “quite a bit,” and 3 for “very much.” We then calculated the average score for each subscale. After receiving a questionnaire, parents rated their children's behavior and behavioral severity within the last 6 months. The completion time for the scale was ~10 min. The SNAP-IV Rating Scale has been demonstrated to have satisfactory validity and reliability in prior research (29).

#### Behavioral Problems

The CPRS is used to assess common behavioral problems in children aged 3–16 years and is mainly implemented in the assessment of children with ADHD. The CPRS consists of five subscales and hyperactivity indices: conduct problems, learning problems, psychosomatic problems, impulsivity-hyperactivity, anxiety, and hyperactivity indices. There are 48 items in the CPRS. Each item is scored on a four-level scale ranging from 0 to 3: “0” indicates that there is no such problem, “1” indicates that there is an occasional or slight performance decrease, “2” indicates frequent or serious behaviors, and “3” indicates very common and/or very serious behaviors. We added the scores of the items contained in each subscale and divided the total score by the number of items in order to obtain subscale-specific scores. The number of CPRS items is moderate, its content is simple and easy to understand, and parents can complete the scale within ~5–10 min. This scale is widely used and is a good assessment tool for children with ADHD (30).

#### Inhibition Control

The SCWT consists of three color-printed cards representing color tasks, word tasks, and combined color–word tasks. Each card consists of 24 words or dots. The first step of the test is to present card A (i.e., the color task), which is composed of dots of four different colors (red, green, yellow, and blue). The second step is to present card B (the word task), which is composed of words in red, green, yellow, and blue (excluding words with color meanings). The third step is to present card C (the color–word task) and represent the four words red, green, yellow, and blue with colors different from their word meanings. The participants were required to correctly read the colors of the dots and words on each card as soon as possible. Evaluators recorded the time it took participants to complete the tasks and the number of mistakes they made in each task. Interference scores indicates the ability to suppress interference. Specifically, time interference is defined as the time necessary to complete a color–word task minus the time to complete the word task; error interference is defined as the number of errors in the color–word task minus the number of errors in the word task.

The go/no-go task used “R” to indicate reactive stimuli (accounting for 80% of stimuli) and “P” to indicate non-reactive stimuli (accounting for 20% of stimuli). At the beginning of the task, the fixation point “+” was shown for 400 ms. The stimulus was then randomly presented at the center of the screen (lasting for 200 ms), with a randomly changing (800 ± 200 ms) inter-stimulus interval. The participants were instructed to press the button as quickly and accurately as possible when they saw “R” and to not press the button when they saw “P.” There were 136 “R” and “44” P stimuli presented during the entirety of the task, and the total completion time was ~5 min. The participants were required to remain quiet in the test environment. Prior to the test, participants were guided with respect to task instructions, with testers explaining the associated requirements and precautions. The recorded indices were the number of missed keys, the number of wrong keys, correct response times, and response time variations (represented by the ratio of the standard deviation of the average response time to the average response time).

#### Working Memory

The WISC-IV is widely used in clinical intelligence tests and presents high reliability and validity (31). The WISC-IV consists of four subscales (verbal comprehension, perceptual reasoning, working memory, and processing speed) as well as a comprehensive full-scale IQ (FSIQ). In the current study, the WMI subscale was used to evaluate working memory ability. The WMI is evaluated based on reciting numbers (also known as digit span) and letter number sequencing subtests. The reciting numbers test requires reciting numbers sequentially and inversely. Sequential reciting refers to the principal tester reading out a sequence of numbers from 2 to 11 (the first level corresponds to two numbers, and each additional level adds one number, a total of 10 levels), with the participants reciting the sequence in the same order. Within this task, when sequence is over, the reverse sequence is started. The principal tester reads a series of numbers from 2 to 9 (level 1 and level 2 correspond to two numbers, and one number is increased for each level from level 3 onward, for a total of nine levels), and the participants recites the numbers in reverse order. The sequence compositions for the sequential and reverse recited numbers are different. If a participant fails to pass the same question twice, the test is terminated. One point was awarded for each pass score for level 1, and no points were deducted for errors. The total score for the reciting numbers task is the sum of the individual scores based on reciting numbers in order and in reverse order. In the letter number sequencing test, the main tester reads a list of numbers and letters (levels 1 and 2 are composed of one letter and one number, levels 3–5 are composed of two letters and one number or one letter and two numbers, and one number or letter is added for each additional level from level 6 onward, a total of 10 levels); the participants recite the numbers they hear from small to large and recite the letters they hear in English alphabetical order. When the participant fails to pass the same question after three attempts, the test is terminated. One point is obtained for each pass for level 1, no points are deducted for errors, and the final score is recorded by the study evaluators. The final WMI is the composite score of the two subtests (reciting numbers and letter–number sequencing).

The n-back test was used to evaluate working memory capabilities *via* three subtasks. At the beginning of the task, the “+” fixation point appears at the center of the computer screen. After 500 ms, a 1 cm × 1 cm gray square appears randomly at the upper left corner, the upper right corner, or the lower right corner, containing the symbol “+” and lasting for 400 ms. The next gray square appears after an interval of 3,000 ms. In the 0-back task, participants were asked to press the left side of the mouse with their right index finger when the square containing the symbol “+” appeared at the upper left corner. Participants were instructed to press the right mouse button with the middle finger of their right hand if the square was to appear in the upper or lower right corner. In the 1-back task, participants were asked to press the right mouse button if the square in the next figure were to appear in the same position as the square in the previous figure and to press the left button if the presentation was different. In the 2-back task, participants were asked to press the right mouse button if the position of the square in the next figure was the same as the square in the previous graph of the previous graph and to press the left mouse button if the presentation was different. There were 30 trials in each task, and the total completion time was ~10 min. The accuracy rate for each task was calculated as the number of gray squares with correct presses divided by the total number of gray squares for each corresponding task.

### Image Acquisition

MRI data were collected using a 3.0 T Siemens Trio Tim scanner (Siemens, Munich, Germany). All participants were asked to close their eyes and to keep their bodies still during the scan. Rs-fMRI data were collected using echo planar imaging [repetition time (TR) = 2,000 ms, echo time (TE) = 30 ms, flip angle (FA) = 90°, matrix size = 94 × 94, field of view (FOV) = 220 mm × 220 mm, volume number = 130, 36 slices, 3-mm slice thickness]. In addition, high-resolution T1-weighted images were acquired using three-dimensional magnetization-prepared rapid gradient echo imaging [TR = 2,000 ms, TE = 2.26 ms, inversion time (TI) = 900 ms, flip angle = 8°, matrix size = 256 × 200, layer number = 176, 1-mm thickness].

### Data Preprocessing

DPABI 4.3 Advanced Edition software (http://rfmri.org/dpabi) based on MATLAB (2014a; MathWorks, Natick, MA, USA) was used to conduct the MRI data preprocessing and associated statistical analyses (32). The processing procedure was as follows: (1) remove first 10 time points; (2) slice timing; (3) head motion correction; (4) nuisance covariate regression (i.e., linear drift, white matter, cerebrospinal fluid); (5) spatial normalization; (6) smoothing (smooth core for 4 mm); and (7) filtering (0.01–0.10 Hz).

### Head Motion Control

Image data can generate information on the mean frame-wise displacement (FD) during scanning. According to Jenkinson's relative root mean square algorithm (33), we excluded participants whose mean FD exceeded 0.2 mm. Four participants who were TT homozygous and three participants who were C-allele carriers were excluded according to this criterion. Head movement effect was controlled by including the mean FD values as covariables within subsequent statistical analyses.

### Regional Homogeneity, Fractional Amplitude Low-Frequency Fluctuation, and Functional Connectivity Calculations

ReHo, fALFF, and FC analyses were performed using DPARSF5.0 Advanced Edition software (http://rfmri.org/DPARSF). ReHo is a voxel-based measure of brain activity that evaluates the similarity or synchronization between the time series of a given voxel and its nearest neighbors. The ReHo was calculated as follows: in order to reduce low-frequency drift and high-frequency noise, we performed a bandpass filter on the spatial standardized data, and Kendall's coefficient of concordance was used to calculate the similarity of the time course between a given voxel and the nearest 26 voxels (34). Next, the ReHo image for each participant was divided by the average ReHo in the brains of all participants in that group. Finally, spatial smoothing (i.e., with a smooth core for 4 mm) was performed on the ReHo brain map.

The fALFF reflects the intensity of regional spontaneous brain activity (35) and was calculated as follows. First, the functional data were preprocessed to obtain the data for linear drift removal. Next, fast Fourier transform was used to transform the time series for each voxel to the frequency domain to obtain the power spectrum. In each voxel, the square root of the power spectrum was calculated at each frequency and was averaged across the entire frequency range. The ratio of the low frequency (0.01–0.08 Hz) power spectrum to the whole frequency range was then calculated. To reduce the global effects of variability across participants, the individual fALFF map was transformed into a Z-score map *via* Fisher-Z transformation.

FC refers to the degree of correlation between the blood oxygenation level-dependent signal sequences in different brain regions within a given time dimension. The FC was calculated as follows: the brain regions with statistically significant differences in ReHo or fALFF between the two groups were defined as regions of interest (ROIs). The mean time series for all voxels in each ROI were then calculated. Pearson's correlation coefficients were used to calculate the FC between the mean time series for each ROI and that of each voxel within the whole brain. Finally, Fisher's Z-transform was used to normalize the correlation coefficients.

### Statistical Analysis

For general demographic and clinical data, statistical analyses were completed using Statistical Package for the Social Sciences (SPSS) software, version 23.0 (Chicago, IL, USA). The measurement data conformed to a normal distribution, and we thus analyzed the differences in age, FSIQ, mean FD, and behavioral performance scores between the two groups using independent sample *t*-tests. The results were expressed as means ± standard deviations. When the resulting *p*-value was very close to 0.05, the effect size was further calculated. Cohen's d was used to measure effect size (36); this statistic was computed by dividing the difference between group means by the pooled standard deviation weighted by the sample size. An effect size of 0.2 corresponds to a small effect, an effect size of 0.5 corresponds to a medium effect, and an effect size of 0.8 corresponds to a large effect.

For MRI data, two sample *t*-tests were performed using DPABI 4.3 Advanced Edition Statistical Analysis to identify brain area differences between the two groups with respect to ReHo, fALFF, and FC. Age, head movement, and the WMI were taken as covariates to exclude any confounding effect on the results. The Gaussian random field (GRF) theory was used for multiple comparisons with voxel *p* < 0.001 and cluster *p* < 0.05 (two-tailed). The GRF controls the thresholds for certain error rates within test statistics in order to improve the accuracy and authenticity of the results (37).

Behavioral indicators with statistically significant differences between the two groups were selected for further correlation analyses. WMI was selected for further analysis in this study. And the fALFF/ReHo/FC clusters showing statistically significant group differences were extracted as ROI masks. The “ROI Signal Extractor” in the Utilities module of the DPABI toolkit was used to extract the time series for each group of ROIs. Finally, SPSS software was used to conduct partial correlation analyses between the ROI time series for each group and the corresponding WMI, controlling for the potentially influencing factors of age and mean FD. The correlations were considered statistically significant when *p*-values were < 0.05.

Finally, controlling for age and mean FD, we calculated the partial correlation analysis between ReHo, fALFF, and working memory ability scores in each group. The GRF was used for correcting multiple comparisons (two-tailed, voxel *p* < 0.001, cluster *p* < 0.05). Time series were extracted for related brain regions, and partial correlation analysis was performed with respect to the WMI, controlling for age and mean FD.

## Results

### General Demographic and Clinical Data

A total of 71 children with ADHD were enrolled in this analysis (TT homozygous group = 34, C-allele carrier group = 37). Age, FSIQ, and mean FD did not differ at the level of statistical significance between the TT homozygous and C-allele carrier groups (*p* > 0.05). With respect to ADHD symptoms, the scores for the TT homozygotes were higher than those of C-allele carriers within the two subscales in the parent version of the SNAP-IV Rating Scale. In addition, we also found that TT homozygotes showed more serious behavioral problems, including inappropriate conduct, impulse hyperactivity, and behavior assessed through the hyperactivity index, as compared with C-allele carriers within univariate analyses. As negative controls, the C-allele carriers showed stronger inhibition capabilities as compared with the TT homozygous group. With respect to working memory ability, we found that the accuracies for TT homozygotes in the 0-back task and in the 1-back task were higher than that of C-allele carriers. In the 2-back task, the accuracies of the two groups were essentially the same. We found a statistically significant difference in the WMI between the two groups within univariate tests, and the *p*-value after conducting multivariate-adjusted statistical analysis was close to 0.05. We further calculated that the effect size was 0.435 (representing a moderate effect), indicating that increasing the sample size could achieve statistical significance. Although children in the TT homozygous group performed worse on clinical behavioral assessment scales as compared to C-allele carriers, the differences were not statistically significant (*p* > 0.05) (Table 1).

**Table 1:** Demographic and clinical characteristics of children with ADHD in the two groups.

	**TT homozygous**	**C-allele carriers**		
	***N*** **= 34**	***N*** **= 37**	**t**	***p***
Age	8.75 ± 0.55	8.88 ± 0.61	−0.913	0.365
Grade	2.82 ± 0.71	2.81 ± 0.77	0.072	0.943
FSIQ	85.76 ± 8.85	86.29 ± 7.09	−0.281	0.780
Mean FD	0.09 ± 0.06	0.08 ± 0.06	0.800	0.426
WMI	83.29 ± 10.21	87.62 ± 9.73	−1.828	0.072
**CPRS**				
Conduct problem	1.21 ± 0.51	1.16 ± 0.49	0.408	0.685
Learning problem	1.91 ± 0.56	1.99 ± 0.66	−0.560	0.578
Psychosomatic disorder	0.27 ± 0.35	0.30 ± 0.31	−0.335	0.739
Impulsivity-Hyperactivity	1.76 ± 0.68	1.54 ± 0.71	1.361	0.178
Anxiety	0.55 ± 0.48	0.73 ± 0.59	−1.389	0.169
Hyperactivity indices	1.64 ± 0.56	1.57 ± 0.53	0.502	0.617
**SNAP-IV**				
SNAP-IA	2.06 ± 0.60	1.92 ± 0.69	0.850	0.398
SNAP-HI	1.67 ± 0.64	1.53 ± 0.61	0.935	0.353
SNAP-ODD	1.29 ± 0.71	1.34 ± 0.61	−0.344	0.732
**SCWT**				
Time interference	19.11 ± 10.52	18.91 ± 11.69	0.074	0.942
Error interference	2.23 ± 2.11	2.18 ± 1.79	0.099	0.921
**Go/no-go Task**				
Number of missed keys	7.52 ± 5.57	7.75 ± 8.35	−0.134	0.894
Number of wrong keys	23.38 ± 5.53	22.75 ± 5.09	0.494	0.623
Correct response time	427.98 ± 81.61	412.06 ± 61.52	0.933	0.354
Response time variation	159.34 ± 44.47	148.97 ± 41.50	1.016	0.313
**N-back Task (correct rate)**				
0-Back	0.86 ± 0.16	0.88 ± 0.10	−0.655	0.514
1-Back	0.60 ± 0.25	0.62 ± 0.19	−0.417	0.678
2-Back	0.41 ± 0.15	0.40 ± 0.16	0.268	0.790

*ADHD, attention deficit/hyperactivity disorder; FSIQ, Full-Scale Intelligence Quotient; FD, framewise displacement; WMI, working memory index; CPRS, Conners' Parent Rating Scale; IA, inattention; HI, hyperactivity/impulsivity; ODD, oppositional defiant disorder; SCWT, Stroop Color Word Test*.

### Regional Homogeneity and Fractional Amplitude Low-Frequency Fluctuation Results

**Figure 1:**
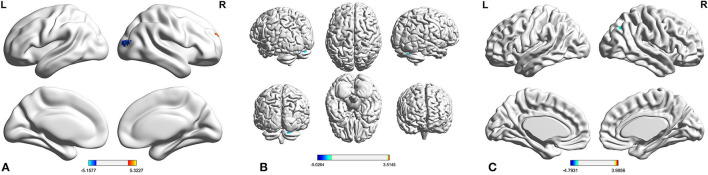
Compared with C-allele carriers, the TT homozygous had decreased ReHo in the right MOG and increased ReHo in the right SFG **(A)**; the TT homozygous had reduced FC in the right MOG and right cerebellum **(B)**, and the TT homozygous had reduced FC in the right SFG and angular **(C)**. ADHD, attention deficit hyperactivity disorder; FC, functional connectivity; MOG, middle occipital gyrus; ReHo, regional homogeneity; SFG, superior frontal gyrus.

Compared with C-allele carriers, TT homozygotes had decreased ReHo in the right middle occipital gyrus (MOG) (Figure 1A; coordinates: 36, −87, 12) and increased ReHo in the right superior frontal gyrus (SFG) (coordinates: 18, 57, 27) (GRF-corrected *p* < 0.05). The fALFF did not differ between TT homozygotes and C-allele carriers at the level of statistical significance.

### Functional Connectivity Results

Considering FC based on the right MOG as a seed, TT homozygotes had reduced FC in the right MOG and the right cerebellum as compared with the C-allele carriers (Figure 1B; coordinates: 21, −75, −24) (GRF-corrected *p* < 0.05). Considering FC based on the right SFG as a seed, TT homozygotes had reduced FC in the right SFG and the angular as compared with the C-allele carriers (Figure 1C; coordinates: 39, −60, −36) (GRF-corrected *p* < 0.05).

### Correlation Analysis

After comparing the behavioral indicators for the two groups, we found a large difference in the WMI between groups. Therefore, we analyzed the correlations between WMI and statistically significantly different brain regions between the groups after controlling for age and head movement (mean FD) factors. No statistically significant correlations between WMI scores and ReHo in the right MOG and in the right SFG were observed when comparing the two groups (TT homozygous, right MOG: *r* = 0.047, *p* = 0.813; right SFG: *r* = −0.243, *p* = 0.213; C-allele carriers, right MOG: *r* = −0.027, *p* = 0.882; right SFG: *r* = 0.192, *p* = 0.292). The FC between the right MOG and the cerebellum and the FC between the SFG and the angular gyrus were not statistically significantly correlated with WMI scores in either group (TT homozygous, MOG and cerebellum: *r* = −0.015, *p* = 0.938; SFG and angular gyrus: *r* = −0.240, *p* = 0.219; C-allele carriers, MOG and cerebellum: *r* = −0.241, *p* = 0.184; SFG and angular gyrus: *r* = −0.123, *p* = 0.503).

**Figure 2:**
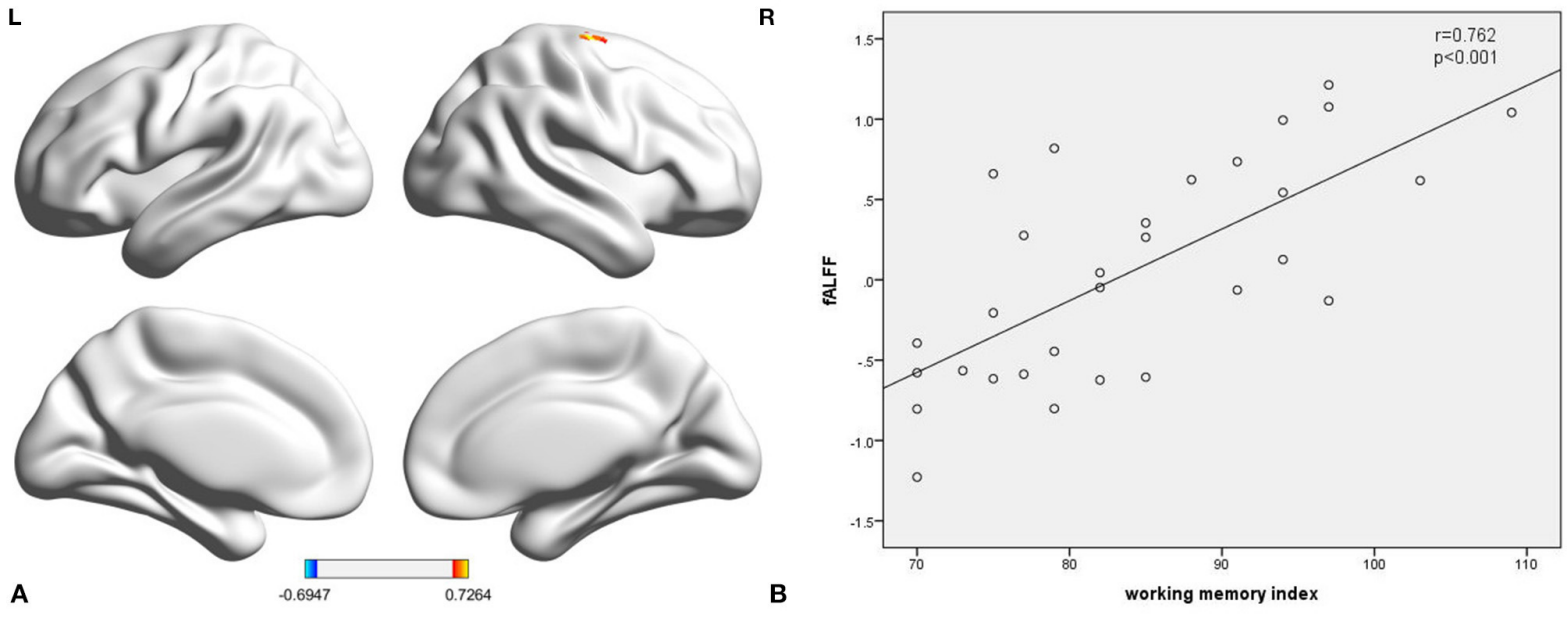
The fALFF of the right precentral gyrus in TT homozygous was significantly correlated with working memory index (*r* = 0.762, *p* < 0.001) **(A,B)**. fALFF, fractional amplitude low-frequency fluctuation.

This study further calculated the association between ReHo, fALFF, and WMI scores in each group. We found a statistically significant positive correlation (Figure 2B; *r* = 0.762, *p* < 0.001) between fALFF in the right precentral gyrus and WMI scores in TT homozygotes (Figure 2A; coordinates: 27, −18, 72) (GRF-corrected *p* < 0.05). There was no statistically significant correlation between fALFF and WMI scores in the C-allele carriers. ReHo was not associated with the WMI in either group.

## Discussion

To our knowledge, this is the first attempt to explore the effects of the *DRD4* −521 C/T SNP on brain function in children with ADHD. The *DRD4* −521 C/T SNP controls the transcription rate for this gene, and its gene expression in turn affects the level of dopamine neurotransmitters in the brain, which is closely related to part of the pathogenesis of ADHD. We observed that compared with C-allele carriers, TT homozygotes showed decreased ReHo in the right MOG in the current study. The occipital lobe is mainly responsible for the processing of visual information and plays an important role in cognitive functions, such as working memory consolidation and attention regulation (38, 39). Sasayama et al. (40) found a decreased volume of gray matter in the bilateral occipital cortex of children with ADHD and the gray matter volume of the right occipital cortex decreased more significantly after controlling for mixed effects such as comorbidities (i.e., oppositional defiant disorder and conduct disorder). It is likely that abnormal structures may be related to lower cognitive function within ADHD. Wang et al. (41) used graph theory analysis to explore changes in the topological structure of the brain functional network in children with ADHD. They found that node efficiency in the occipital cortex was statistically significantly reduced among children with ADHD. In addition, in a 33-year longitudinal follow-up study, adult patients with persistent ADHD in childhood had statistically significantly less occipital cortex thickness as compared with adults without ADHD in childhood (42). A study based on rs-fMRI found that FC in the occipital cortex was decreased in ADHD patients, and that FC was negatively correlated with attention deficit scores (43). It was hypothesized that the decreased ReHo in the right MOG among TT homozygotes may increase the severity of their core symptoms to some extent, and that TT homozygotes may thus show more severe cognitive deficits as compared to C-allele carriers.

We also found that the TT homozygotes increased ReHo in the right SFG. Abnormal frontal lobe function is an important cause of executive dysfunction in children with ADHD (44). Dopamine neurotransmitters are mainly expressed in the prefrontal cortex and regulate changes in neuronal activity to facilitate the accurate performance of cognitive tasks (45). Peterson et al. (46) adopted the method of diffusion tensor imaging and found that, compared with normal children, the fractional anisotropy among ADHD children increased at the level of statistical significance; this effect was mainly concentrated in the right SFG. The observed increase in fractional anisotropy is closely related to the severity of ADHD symptoms. Wang et al. (47) analyzed differences in local spatiotemporal consistency between children with ADHD and neurotypical children and found that the four-dimensional (spatiotemporal) consistency of local neural activities (FOCA) in the right SFG increased among children with mixed ADHD. Ma et al. (48) used event-related fMRI to study neural responses among children with ADHD and neurotypical controls with respect to reward SCWT scores. These researchers found that reward signals among children with ADHD within the right SFG increased as compared with the control group. It is speculated that abnormal activity in the right SFG may be one of the principal mechanisms leading to deficiencies in executive function observed among children with the TT homozygous genotype.

We performed seed-based FC studies and found that, in TT homozygotes, the FC between the right MOG and the cerebellum decreased and the FC between the right SFG and angular gyrus decreased. This indicates that TT homozygotes have a weaker brain FC network. The cerebellum plays an important role in cognition and emotion as well as in motor learning and coordination (49). Previous studies have found abnormal functional activity of the cerebellum among children with ADHD and that the cerebellum is an important brain region for ADHD with regard to executive function defects (50, 51). Some scholars have found that children with ADHD have lower long-range FC density in the cerebellum as compared with a typical developing child (52). In addition, Goetz et al. (53) found that the cerebellar symptom scores of children with ADHD decreased with age, while those of normal children remained stable. Furthermore, the cerebellar symptom scores were associated with omission errors, overall response time standard error, and prolonged stimulation intervals.

The angular gyrus plays an important role in semantic processing, word reading comprehension, number processing, memory retrieval, spatial cognition, and reasoning (54). Previous studies have found that the temporal variability of the angular gyrus is statistically significantly increased in children with ADHD (55). Compared with typically developing children, children with ADHD have statistically significantly reduced activation in the angular gyrus, which is in turn related to abilities with respect to goal-directed behavior and attention regulation (56). However, in the current study, we did not find a relationship between the WMI and brain area-specific activation. This may be because only a single WMI was selected for correlation analysis in the current study, there were no statistically significant difference between WMI for the two groups, and the sample size within this study was relatively small, limiting our statistical power to detect associations.

We compared fALFF between the two groups and found no statistically significant differences in brain regions. Our results can be explained as follows. First, both fALFF and ReHo reflect the spontaneous activity of local neurons, but the specific mechanisms mediating these effects differ. Specifically, ReHo values describe the synchronization of the activity of adjacent voxel neurons, while fALFF describes the intensity of neuron activity at the voxel level. It is likely that there were no statistically significant differences in the intensity of local neuronal activity between the two groups. Second, some scholars have proposed that ReHo can more sensitively reflect different brain functional activities as compared with fALFF (57). In addition, this study calculated correlations between ReHo, fALFF, and working memory scores in each group. We found a statistically significant positive correlation between fALFF in the right precentral gyrus and the WMI in TT homozygotes. The precentral gyrus belongs to the sensorimotor cortex and plays an important role in controlling verbal thinking, planning goal orientation, and adjusting volitional activities to ensure correct purposeful behavior. Previous studies have shown that the thickness of the precentral gyrus among children with ADHD is statistically significantly thinner than that of healthy children (58). In addition, other studies have found that the gray matter volume in the right precentral gyrus among children with ADHD is statistically significantly reduced as compared with neurotypical children (59). Our results show that the right precentral gyrus among children with ADHD who are TT homozygous shows a lower working memory ability with an accompanying decrease in fALFF. There was no statistically significant correlation between fALFF and the WMI in C-allele carriers. These previous findings support the potential link between working memory ability and fALFF within the right precentral gyrus, as observed in the current study.

In addition to the substantial strengths of this investigation. Our study has some limitations, and a larger sample is needed in the future to determine the robustness of the results. First, the inclusion criteria were strict, such that only boys with ADHD, without psychotropic drug treatment, and without any other comorbidities were enrolled in the current study; these strict inclusion and exclusion criteria restrict the generalization of our findings with respect to the entire ADHD population. Second, the differences between children with TT homozygous and C-allele carriers were not statistically significant with regard to behavioral assessment scores; this was probably due to the modest sample size of the current study, with resulting low statistical power. Third, the partial correlation analyses between ReHo/FC and working memory abilities in each group did not show statistically significant correlations. This finding may be explained by the vague boundedness of the selected behavior indicators. Future studies need to examine a wider range of behavioral indicators within correlation analyses. Fourth, the small sample size in our study limited the scope and power of ADHD subtype analyses for each group. Notably, this study attempted to be pioneering with regard to enrolling children with ADHD and examining the effects of *DRD4* −521 C/T polymorphisms *via* resting-state brain fMRI. Therefore, little existing evidence is available to support the findings of the current study.

In summary, our findings support our hypothesis that the *DRD4* −521 C/T SNP has different effects on local brain activity and FC in children with ADHD. The results of this study suggest that children with ADHD with TT homozygous genotypes may suffer from more salient brain dysfunction, which is consistent with the maladaptive behaviors observed among TT homozygotes. Due to the limitations of our study, the effects found need to be replicated first, and larger samples will be needed in the future to understand the robustness of the results.

## Data Availability Statement

The raw data supporting the conclusions of this article will be made available by the authors, without undue reservation.

## Ethics Statement

The studies involving human participants were reviewed and approved by Medical Research Ethics Committee of Shenzhen Children's Hospital. Written informed consent to participate in this study was provided by the participants' legal guardian/next of kin.

## Author Contributions

HZ and BY conceived and designed the experiments, analyzed the data, and performed the statistical analysis. GP, LZ, and DF collected the data. HZ drafted the article with critical comments from BY. All authors contributed to the article and approved the submitted version.

## Funding

This work was supported by the Natural Science Foundation of China (Grant No. 81271512) and the Sanming Project of Medicine in Shenzhen (SZSM 201612036).

## Conflict of Interest

The authors declare that the research was conducted in the absence of any commercial or financial relationships that could be construed as a potential conflict of interest.

## Publisher's Note

All claims expressed in this article are solely those of the authors and do not necessarily represent those of their affiliated organizations, or those of the publisher, the editors and the reviewers. Any product that may be evaluated in this article, or claim that may be made by its manufacturer, is not guaranteed or endorsed by the publisher.
